# Stereotactic Comparison Study of ^18^F-Alfatide and ^18^F-FDG PET Imaging in an LLC Tumor-Bearing C57BL/6 Mouse Model

**DOI:** 10.1038/srep28757

**Published:** 2016-06-28

**Authors:** Yu-Chun Wei, Yongsheng Gao, Jianbo Zhang, Zheng Fu, Jinsong Zheng, Ning Liu, Xudong Hu, Wenhong Hou, Jinming Yu, Shuanghu Yuan

**Affiliations:** 1 Department of Radiation Oncology, Shandong Cancer Hospital Affiliated to Shandong University, Jinan, China; 2 School of Medicine and Life Sciences, University of Jinan-Shandong Academy of Medical Sciences, Jinan, China; 3 Department of Pathology, Shandong Cancer Hospital Affiliated to Shandong University, Jinan, China; 4 Department of Nuclear Medicine, Shandong Cancer Hospital Affiliated to Shandong University, Jinan, China

## Abstract

This study aimed to stereotactically compare the PET imaging performance of ^18^F-Alfatide (^18^F-ALF-NOTA-PRGD_2_, denoted as ^18^F-Alfatide) and ^18^F-fluorodeoxyglucose (FDG) and immunohistochemistry (IHC) staining in Lewis lung carcinoma (LLC) tumor-bearing C57BL/6 mouse model. ^18^F-FDG standard uptake values (SUVs) were higher than ^18^F-Alfatide SUVs in tumors, most of the normal tissues and organs except for the bladder. Tumor-to-brain, tumor-to-lung, and tumor-to-heart ratios of ^18^F-Alfatide PET were significantly higher than those of ^18^F-FDG PET (*P* < 0.001). The spatial heterogeneity of the tumors was detected, and the tracer accumulation enhanced from the outer layer to the inner layer consistently using the two tracers. The parameters of the tumors were significantly correlated with each other between ^18^F-FDG SUV and GLUT-1 (R = 0.895, P < 0.001), ^18^F-Alfatide SUV and αvβ3 (R = 0.595, P = 0.019), ^18^F-FDG SUV and ^18^F-Alfatide SUV (R = 0.917, P < 0.001), and GLUT-1 and αvβ3 (R = 0.637, P = 0.011). Therefore, ^18^F-Alfatide PET may be an effective tracer for tumor detection, spatial heterogeneity imaging and an alternative supplement to ^18^F-FDG PET, particularly for patients with enhanced characteristics in the brain, chest tumors or diabetes, meriting further study.

Positron emission tomography (PET), which uses radiolabeled tracers, is a promising, noninvasive imaging method for a more accurate evaluation of initial staging, regional and distant metastasis, treatment response, and recurrence^1^,^2^. ^18^F-fluorodeoxyglucose (FDG), a commonly used tracer for neoplasm detection, and also a marker of glycolytic metabolism, is widely used in the clinical field. Increased expression of glucose transporter (GLUT) proteins is the foundation of the standard uptake values (SUVs) of FDG positron emission tomography (PET)^3^. GLUT-1 is one of the carriers of the GLUT family. GLUT-1 expression is undetectable in most normal epithelia and benign epithelial tumors, and its expression has been shown to indicate epithelial malignancy in various organs^4^. Metabolic imaging with ^18^F-FDG PET and computed tomography (CT) is known to be a useful method in evaluating tumors^5^. However, ^18^F-FDG PET has its own limitation in differentiating tumors in some special organs with a high background, such as the brain, heart, kidney, and bladder, and some diabetes patients. Therefore, many new tracers have been developed as supplements to FDG.

RGD peptide with a sequence of the three amino acids Arg-Gly-Asp is an integrin αvβ3-specific ligand. Integrin αvβ3 is significantly up-regulated in various types of tumor cells and in the activated endothelial cells of tumor angiogenesis but is not up-regulated at all or is only slightly up-regulated in quiescent vessel cells and other normal cells^6^. Integrin αvβ3 has been intensively investigated as a target for angiogenesis imaging and therapy^7^,^8^. Therefore, radiolabeled RGD has been used extensively for PET imaging tumor angiogenesis^9^,^10^. Currently, several ^18^F-labeled RGD peptides, including ^18^F-galacto-RGD^11^, ^18^F-AH111585 12, ^18^F-RGD-K5 13, ^18^F-FPRGD2 and ^18^F-FPPRGD2, have been evaluated in clinical trials^14^. However, the multiple-step synthetic procedure for the preparation of these ^18^F-labeled RGD tracers is very time-consuming^15^, with a relatively low labeling yield, possibly hindering their widespread use. The easy-to-prepare and high-yield qualities make [(18) F]AIF-NOTA-PRGD2, denoted as ^18^F-Alfatide, a promising alternative for PET imaging of tumor αvβ3 integrin expression^16^,^17^.

In this study, we performed micro-PET imaging using both ^18^F-alfatide and ^18^F-FDG in an LLC tumor-bearing mouse model. We aimed to stereotactically compare the PET imaging performance and standardized uptake value of ^18^F-Alfatide and ^18^F-FDG, and also used immunohistochemistry (IHC) staining in a Lewis lung carcinoma (LLC) tumor-bearing C57BL/6 mice model.

## Results

### Biodistribution Data by PET Imaging

A total of 15 C57BL/6 mice were transplanted with LLC in the right thigh successfully, and then they underwent PET imaging. During imaging, no significant adverse events were observed. The radiotracer biodistribution was measured in major organs at 1 hour after injection of ^18^F-Alfatide and ^18^F-FDG (Table 1). The highest accumulation activity was found in the kidneys and bladder with both radiotracers, demonstrating renal clearance. The blood also showed moderate uptake with the two radiotracers PET imaging. High radiotracer accumulation was found in the brain, heart, lung, and muscle in ^18^F-FDG PET scans, whereas those of ^18^F-Alfatide PET were minimal.

### Tumor Detection

Fifteen xenografts were all detected on both ^18^F- FDG PET and ^18^F-Alfatide PET. The results of the SUV measurements for tumors assessed by ^18^F-Alfatide (SUV_RGD_, 2.37 ± 0.46) was lower than ^18^F-FDG (SUV_FDG_, 10.19 ± 1.67), (*P* < 0.001).

Table 2 shows that, although the ^18^F-FDG mean SUVs were higher than ^18^F-Alfatide mean SUVs in tumors and most of the normal tissues and organs, the tumor-to-brain, tumor-to-lung, and tumor-to-heart ratios of ^18^F-Alfatide PET were significantly higher than those of ^18^F-FDG PET (*P* < 0.001). The tumor-to-brain, tumor-to-heart, tumor-to-lung, and tumor-to-muscle ratios of ^18^F-Alfatide were 5.22 ± 1.83, 3.68 ± 0.67, 5.28 ± 0.92, and 4.64 ± 1.49, respectively, whereas those of ^18^F-FDG PET were 2.33 ± 0.46, 1.00 ± 0.29, 2.38 ± 0.77, and 5.27 ± 1.70, respectively. The *P* values were <0.001, <0.001, <0.001, and =0.365, respectively.

### Spatial Heterogeneity Imaging

**Figure 1:**
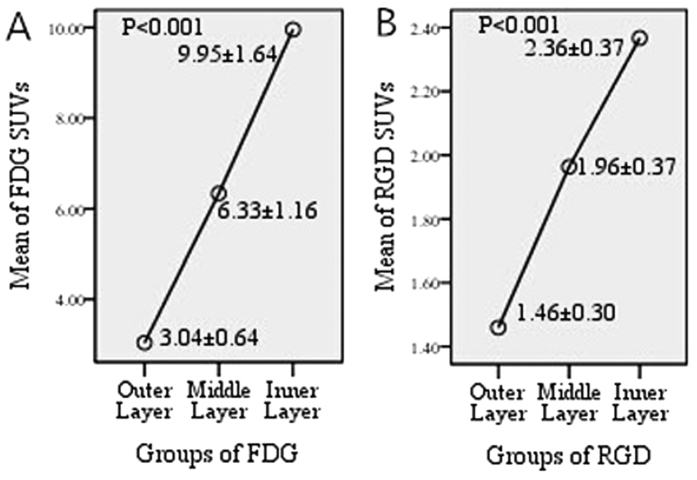
SUVs of outer, middle, and inner layers for FDG (**A**) scans and RGD (**B)** scans in xenografts. The tracers accumulation enhanced from out layer to inner layer consistently on the two tracers (SUV_FDG-O_, SUV_FDG-M_, and SUV_FDG-I_ were 3.04 ± 0.64, 6.33 ± 1.16, 9.95 ± 1.64 and SUV_RGD-O_, SUV_RGD-M_, and SUV_RGD-I_ were 1.46 ± 0.30, 1.96 ± 0.37, 2.36 ± 0.37, P < 0.001).

The spatial heterogeneity of the xenografts was detected, and the radiotracer accumulation was enhanced from the outer layer to the inner layer consistently using the two tracers. Figure 1 shows the SUV of the outer (SUV_RGD-O_, SUV_FDG-O_), middle (SUV_RGD-M_, SUV_FDG-M_) and inner (SUV_RGD-I_, SUV_FDG-I_) layers by ^18^F-Alfatide and ^18^F-FDG PET. SUV_FDG-O_, SUV_FDG-M_, and SUV_FDG-I_ were increased in turn (3.04 ± 0.64, 6.33 ± 1.16, 9.95 ± 1.64, respectively, *P* < 0.001), as well as SUV_RGD-O_, SUV_RGD-M_, and SUV_RGD-I_ (1.46 ± 0.30, 1.96 ± 0.37, 2.36 ± 0.37, respectively, *P* < 0.001).

**Figure 2:**
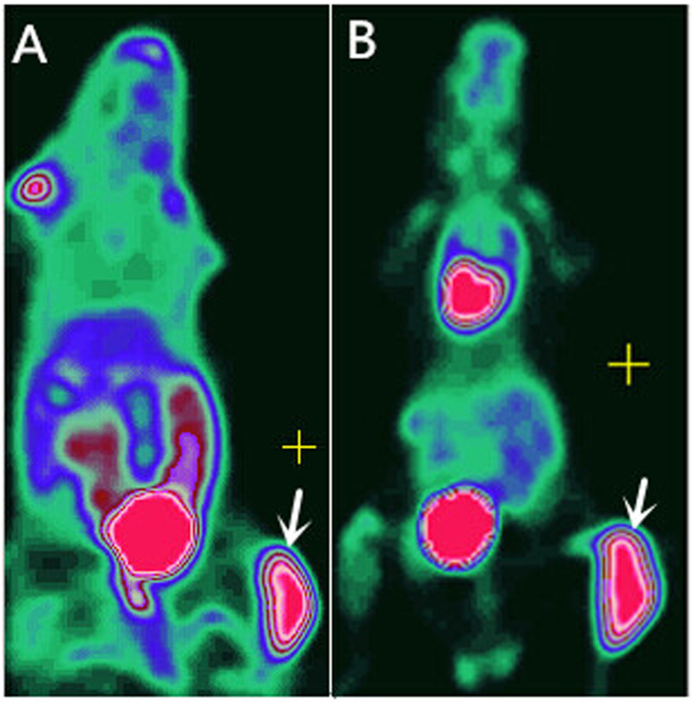
Biodistribution by whole body PET Imaging. Figure 2A, ^18^F-Alfatide PETimaging. Figure 2B, ^18^F-FDG PET imaging. Arrows pont to tumor.

The SUV comparison results by layer are summarized in Table 3 and Fig. 2. SUV_FDG-O_ vs SUV_FDG-M_, SUV_FDG-O_ vs SUV_FDG-I_, SUV_FDG-M_ vs SUV_FDG-I_, SUV_RGD-O_ vs SUV_RGD-M_, SUV_RGD-O_ vs SUV_RGD-I_, and SUV_RGD-M_ vs SUV_RGD-I_ showed significant differences between each other, and *P* values were <0.001, <0.001, <0.001, <0.001, <0.001, and =0.003, respectively.

### Immunohistochemical Validations and their Correlation with PET Imaging

**Figure 3:**
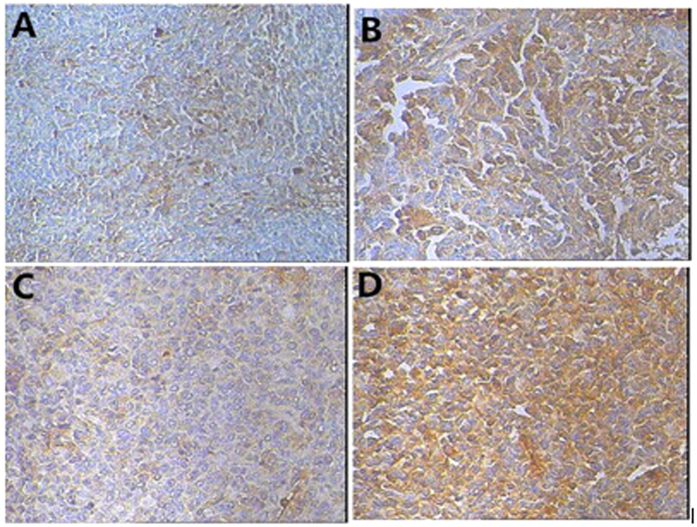
Patterns of αvβ3 and GLUT-1 immunochemistry. Figure 4A, Weak αvβ3 staining (200×). Figure 4B, Strong αvβ3 staining (200×). Figure 4C, Weak GLUT-1 staining (200×). Figure 4D, Strong GLUT-1 staining (200×).

**Figure 4:**
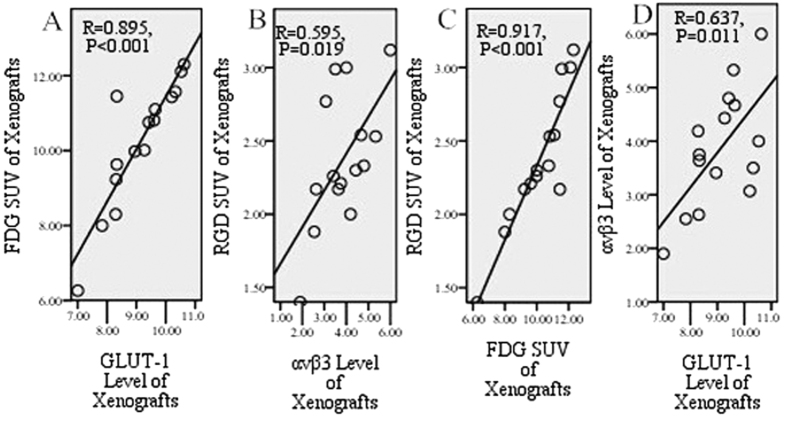
Correlation between SUVs and immunohistochemistry in xenograft. Between FDG SUV and GLUT-1 (R = 0.895, P < 0.001), RGD SUV and αvβ3 (R = 0.595, P = 0.019), FDG SUV and RGD SUV (R = 0.917, P < 0.001), and GLUT-1 and αvβ3 (R = 0.637, P = 0.011) showed significantly positive correlation in xenografts.

PET imaging was validated by IHC examination. The staining of both GLUT-1 and αvβ3 was mainly cytoplasmic (Fig. 3). The expression levels were 9.11 ± 1.08 for GLUT-1 and 3.86 ± 1.10 for αvβ3. Figure 4 shows a positive correlation between SUV_FDG_ and the GLUT-1 expression level (R = 0.895, *P* < 0.001), and between SUV_RGD_ and the αvβ3 expression level in tumors (R = 0.595, *P* = 0.019).

In contrast, Fig. 4C shows a very strong positive correlation between SUV_FDG_ and SUV_RGD_ (R = 0.917, *P* < 0.001). A strong positive correlation was also found between the GLUT-1 expression level and αvβ3 expression level (R = 0.637, *P* = 0.011) (Fig. 4D). These correlation studies showed that the tumor SUV changes of ^18^F-FDG and ^18^F-Alfatide are consistent with each other, as well as with GLUT-1 and αvβ3 expression in rough calculation.

## Discussion

In this study, ^18^F-Alfatide was shown to be a potentially effective tracer for the detection of brain, lung, and heart tumors with higher tumor-to-background ratios than ^18^F-FDG in these organs. All xenografts were identified with high tumor-to-muscle ratios by both ^18^F-Alfatide PET and ^18^F-FDG PET imaging. ^18^F-Alfatide PET and ^18^F-FDG PET imaging were interrelated in tumor detection; SUV_FDG_, SUV_RGD_, GLUT-1, and αvβ3 in tumors were correlated closely with each other, and the spatial heterogeneity of SUV_FDG_ in different tumor layers was consistent with SUV_RGD_ in rough calculation.

^18^F-Alfatide, as a new RGD PET tracer, showed potential advantages for brain and chest tumors because of the high tumor-to-background ratio *in vivo* PET imaging. This finding was consistent with that in prior studies. In the application of lung cancer, Chen X *et al*. found that the primary lung boundary effects of RGD PET imaging is similar to FDG PET, and RGD PET provides better imaging for mediastinal lymph nodes and contralateral lung metastases^18^. Hiroshi Fushiki^19^ *et al*. also reported that ^18^F-FDG was specifically accumulated in tumors and the heart in the thoracic cavity, and there were some high background signals with ^18^F-FDG PET in the chest, including the heart and skeletal muscle around the lung, indicating that ^18^F-FDG PET showed difficulty in recognizing the tumor. In our study, the same tumor-bearing mice imaged with ^18^F-FDG not only showed a high uptake in chest tissues but also in the brain, thus making it difficult to delineate tumor metastases due to low tumor contrast; however, high tumor-to-background ratios exist in those tissues with ^18^F-Alfatide PET images.

Spatial heterogeneity in tumors was found in our study because the tracer accumulation was enhanced from the outer layer to the inner layer consistently with the two tracers. Tumor heterogeneity has been intensively investigated as a target to better serve tumor-individualized treatment. Different PET SUVs in different tumor areas represent different level of glycometabolism and angiogenesis and deserve different radiation dose for tumor control. This has been used in some clinical trial (For example, RTOG1106). Metz^20^ *et al*. conducted a multi-image assessment of non-small cell lung cancer and other cancer patients, and simultaneously underwent RGD PET imaging for angiogenesis and FDG PET imaging for glucose metabolism. They found that the highest perfusion tumor sub-region was also the region with the highest αvβ3 integrin expression (i.e., angiogenesis) and the highest glucose metabolism, and the hypoperfusion area was also the region with low αvβ3 integrin expression and low glucose metabolism. Thus, this multi-mode imaging assessment for tumor heterogeneity is feasible, and this integrated multi-mode imaging can compensate for the lack of a single image. PET imaging would be a convenient method for noninvasive intratumor heterogeneity imaging and individualized radiotherapy. However, radiotracers’ uptakes alone are not necessarily the only factor to consider in terms of tumor heterogeneity. Furthermore, clinical situations are far more complicated than the animal models. Whether spatial heterogeneity on PET imaging has correlation with tumor heterogeneity is still unclear and it deserves further study.

Hyperglycemia is associated with decreased FDG uptake by tumors as assessed by PET^21^. ^18^F-Alfatide PET imaging is based on the expression level of αvβ3, regardless of the glucose metabolism; thus, we believe that ^18^F-Alfatide PET should have more advantages than ^18^F-FDG PET in uncontrolled diabetic patients.

Many studies^22^,^23^,^24^ have shown that ^18^F-FDG uptake values have a strong relationship with the GLUT-1 IHC results. In patients with cervical^25^, ovarian^26^, and endometrial cancer^27^, it has been reported that a positive correlation exists between the SUV of the primary tumor and expression of GLUT-1. GLUT-1 expression, also related to tumor radioresistance at clinically relevant levels, has been reported in several studies^28^. Thus, FDG PET/CT is sensitive in detecting changes in tumor activity after treatment compared with conventional imaging methods^29^. In the current study, a strong positive correlation was found not only between FDG SUVs and GLUT-1 expression in tumors but also between ^18^F-FDG SUV and ^18^F-Alfatide SUV. The latter finding indicated that noninvasive ^18^F-Alfatide PET imaging may play a similar role in evaluating tumor invasion, staging, or detecting changes in tumor activity after treatment using ^18^F-FDG PET.

To further verify the conclusion as mentioned above, we explored the correlation between ^18^F-Alfatide SUV and αvβ3 IHC staining, and the correlation between IHC staining between αvβ3 and GLUT-1. High ^18^F-Alfatide uptake is due to high integrin expression in normal tissues and organs and consequently minimal nonspecific cardiac and lung activity accumulation with this radiotracer. In the current study, a significant positive correlation was found between ^18^F-Alfatide SUV and αvβ3 (R = 0.595, P = 0.019). Consequently, various radiolabeled RGD peptides have been developed for the noninvasive determination of αvβ3 expression^30^. The most extensive use of RGD PET is the monitoring of tumor angiogenesis and anti-angiogenesis therapy^9^,^10^. In this study, a strong positive correlation was also found between GLUT-1 and αvβ3 (R = 0.637, P = 0.011). Combined with the conclusions above, this study may further expand the application of ^18^F-Alfatide PET in detecting tumor growth and monitoring tumor therapy effects compared with ^18^F-FDG PET.

## Conclusion

^18^F-Alfatide PET may be an effective tracer for tumor detection, spatial heterogeneity imaging and an alternative supplement to ^18^F-FDG PET, particularly for patients with enhanced characteristics with brain and chest tumors or diabetes, meriting further study. Although this preclinical study was performed successfully, the application value of ^18^F-Alfatide PET and FDG PET needs further discussion.

## Materials and Methods

### Cell Culture and Animal Tumor Model Preparation

Murine LLC cells, recently used in several high-profile preclinical studies^31^,^32^, were purchased from the Type Culture Collection of the Chinese Academy of Sciences, Shanghai, China. LLC cells were grown in RPMI 1640 (Sigma Chemicals Aldrich, Milan, Italy), supplemented with 10% fetal bovine serum and 1% penicillin streptomycin antibiotic mixture (Life Technologies, Inc.-Invitrogen, Grand Island, NY) in a humidified incubator (Heraeus, Hanau, Germany) at 37 °C with 5% CO_2_ atmosphere. The LLC tumor model was generated by subcutaneous injection of 2 × 10^6^ cells into the right hind leg of C57BL/6 mice (Charles River Lab). Animals were housed in a limited-access animal facility. The animal room temperature and relative humidity were set at 22 ± 2 °C and 55 ± 10%, respectively. Artificial lighting provided a 24-h cycle of 12-h light/12-h darkness (7 a.m. to 7 p.m.).

All animal procedures were approved by the Shandong Cancer Hospital & Institute Ethical Committee Guide for the care and use of Laboratory Animals. The methods were carried out in accordance with the approved guidelines.

### PET Imaging

The mice were subjected to PET studies when the tumor diameter reached approximately 1 cm. The simple lyophilized kit for labeling the PRGD2 peptide was purchased from the Jiangsu Institute of Nuclear Medicine, and the synthesis process was carried out in accordance with previous studies^33^. The radiochemical purity of the ^18^F-Alfatide exceeded 95%, and its specific radioactivity exceeded 37 GBq (1,000 mCi)/μmol. PET data acquisition was performed using an Inveon microPET scanner (Siemens Medical Solutions USA, Inc). With the assistance of the Inveon system’s positioning laser, each LLC tumor-bearing mouse was placed with its tumor located at the center of the field of view, where the highest imaging sensitivity can be achieved.

^18^F-FDG and ^18^F-Alfatide images were performed 1 hour after tail-vein injection under isoflurane anesthesia. Each mouse underwent ^18^F-FDG (2.6–3.6 MBq) PET within 2 days of the ^18^F-Alfatide (2.4–3.5 MBq) PET scan. Before ^18^F-Alfatide PET scanning, no specific subject preparation was applied, and the mice did not need fasting. Before the ^18^F-FDG PET examinations, each mouse had been fasted for at least 4 hours. During the acquisition period, a thermostat-controlled thermal heater maintained the body temperature of the mice. PET emission images were taken from the head to the tail. The images were reconstructed using a 2-dimensional ordered-subsets expectation maximization algorithm. The 10-min static PET scans were then acquired at 1 hour after injection.

### Image Analysis

**Figure 5:**
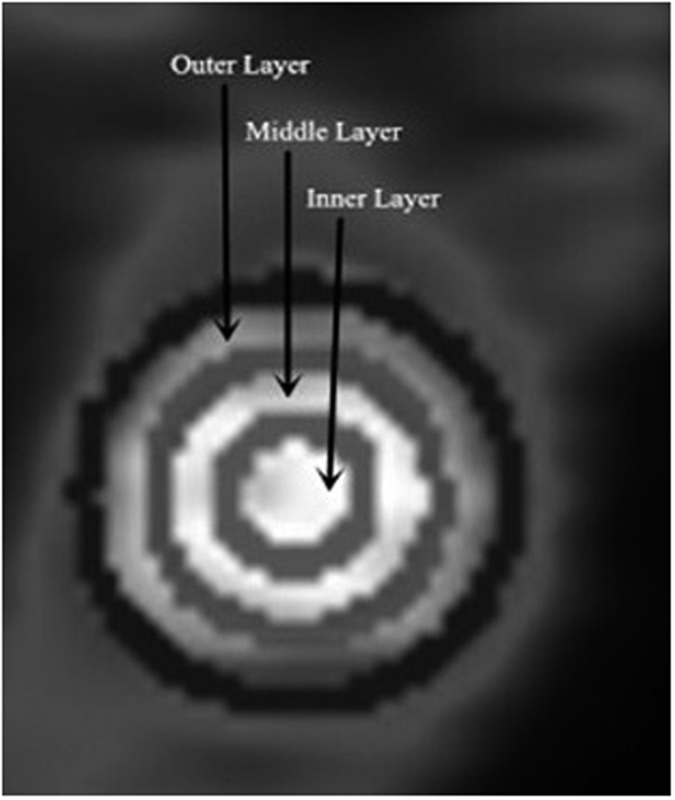
Tumor PET images in coronal plane. Tumor on this slice was roughly divided into outer, middle and inner layers based on the luminance signal.

For each PET scan, regions of interest were drawn over the tumor using vendor software (IS_v1.4.3 SP1; Siemens Medical Solutions) on decay-corrected whole-body coronal images slice by slice. Two experienced nuclear medicine physicians read all of the images through consensus reading. PET images in the tumor were first evaluated by visual analysis, and then a quantitative analysis was performed by determining the SUV. Every of the outlined slices for each tumor was divided into outer, middle, and inner layers based on the luminance signal in the coronal plane by the images (Fig. 5). Then ROI was positioned around the tumor area of interest slice by slice and obtained a set of data such as ROImax, mean. The SUVs were calculated according to the following formula: [measured activity concentration (Bq/mL) × body weight (g)]/injected activity (Bq).

### Immunohistochemistry

The mice were sacrificed to harvest the whole xenografts within 1 day after their micro-PET scans and processed routinely for integrin αvβ3 and GLUT-1 IHC. In all cases, each tumor sample was fixed in 10% formalin and embedded paraffin. Each tumor sample was sectioned sequentially and transversely with a macrotome (Microm HM 450; GMI, Ramsey, MN) into approximately 3- to 5-μm-thick 5 slices at 0.6-mm intervals. One slice was stained with HE, and the others were used for IHC studies. The expression levels of αvβ3 and GLUT-1 were detected by IHC (pv-6000 two-step) (Zhongshan Golden Bridge Biotechnology Corporation, Beijing, China). Integrin αvβ3 (1:400, Sigma) and GLUT-1 (1:250, Abcam) were used as primary antibodies. A section of normal leg muscle was used as the positive control, and negative controls were obtained by omitting the primary antibody.

Integrin αvβ3-positive cells were stained with brown-yellow granules or masses, specifically in the cytoplasm. GLUT-1-positive expression was observed mainly in the cytoplasm. Both the intensity and percentage of positive cells were measured. The staining intensity was determined using the following four classes: 0 = undetectable; 1 = faint buff; 2 = moderate buff; and 3 = high buff or sepia. Stained cell sections in each case were randomly selected, and five high-power fields were counted under the microscope up to 400. We counted 200 cells in each region for a total number of 1,000 cells, calculated the percentage of positive cells, and then calculated the score. Using the percentage of stained cells × staining intensity, the integrated scoring was assessed. Cell expression was stratified as follows: 0 (negative) for no immunoreactivity, 1 for less than 25% positive cells, 2 for less than 50% positive cells, 3 for less than 75% positive cells, and 4 for more than 75% positive cells. The product of the staining intensity and positive cell scores determined the final result for each section.

### Statistical Analysis

All quantitative data are expressed as the mean ± standard deviation (SD). Differences between continuous variables and dichotomous variables were tested by one-way ANOVA. Student’s paired t-test was used to detect differences between the two sample means. Multiple comparisons were compared using LSD. Linear regression analysis was used to evaluate the correlation studies. All statistical tests were carried out using SPSS, version 17.0. Statistical significance was assumed for P values less than 0.05. All *P* values were 2-tailed.

## Additional Information

**How to cite this article**: Wei, Y.-C. *et al*. Stereotactic Comparison Study of ^18^F-Alfatide and ^18^F-FDG PET Imaging in an LLC Tumor-Bearing C57BL/6 Mouse Model. *Sci. Rep.* **6**, 28757; doi: 10.1038/srep28757 (2016).

## Figures and Tables

**Table 1:** Mean SUVs (mean ± SD) Normalized to Weight for Various Organs by ^18^F-Alfatide PET and ^18^F-FDG PET Respectively (n = 15).

**Organ**	^**18**^**F-Alfatide PET**	^**18**^**F-FDG PET**
Blood	1.00 ± 0.16	2.00 ± 0.61
Brain	0.47 ± 0.70	2.16 ± 0.54
Heart	0.44 ± 0.07	5.20 ± 1.40
Lung	0.30 ± 0.04	2.20 ± 0.62
Muscle	0.38 ± 0.14	1.04 ± 0.41
Bladder	14.81 ± 3.73	9.18 ± 3.17
Kidneys	1.48 ± 0.34	2.17 ± 0.59

**Table 2:** Mean SUVs (mean ± SD) Normalized to Weight for Tumor and Tumor-to-Background Ratios by ^18^F-Alfatide PET and ^18^F-FDG PET Respectively (n = 15).

**Organ**	^**18**^**F-Alfatide PET**	^**18**^**F-FDG PET**	**P Value**
Tumor	1.59 ± 0.26	4.94 ± 1.01	
Tumor-to-Blood	1.59 ± 0.24	2.68 ± 1.00	P = 0.002
Tumor-to-Brain	5.22 ± 1.83	2.33 ± 0.46	P < 0.001
Tumor-to-Heart	3.68 ± 0.67	1.00 ± 0.29	P < 0.001
Tumor-to-Lung	5.28 ± 0.92	2.38 ± 0.77	P < 0.001
Tumor-to-Muscle	4.64 ± 1.49	5.27 ± 1.70	P = 0.365
Tumor-to-Bladder	0.11 ± 0.04	1.10 ± 0.19	P < 0.001
Tumor-to-Kidneys	0.60 ± 0.23	2.33 ± 0.45	P < 0.001

**Table 3:** Multiple comparisons of SUV between different layers of tumor (n = 15).

	**P value**
^18^F-Alfatide SUV	
Outer VS. Middle layer	P < 0.001
Outer VS. Inner layer	P < 0.001
Middle VS. Inner layer	P = 0.003
^18^F-FDG SUV	
Outer VS. Middle layer	P < 0.001
Outer VS. Inner layer	P < 0.001
Middle VS. Inner layer	P < 0.001

## References

[b1] Kruse M., Sherry S. J., Paidpally V., Mercier G. & Subramaniam R. M. FDG PET/CT in the management of primary pleural tumors and pleural metastases. AJR Am J Roentgenol 201, W215–226 (2013). 23883236 10.2214/AJR.13.10572

[b2] Histed S. N. . Review of functional/anatomical imaging in oncology. Nucl Med. Commun 33, 349–361 (2012). 22314804 10.1097/MNM.0b013e32834ec8a5 PMC3295905

[b3] Schillaci O. Use of dual-point fluorodeoxyglucose imaging to enhance sensitivity and specificity. Semin Nucl Med. 42, 267–280 (2012). 22681676 10.1053/j.semnuclmed.2012.02.003

[b4] Sasaki H. . Overexpression of GLUT1 correlates with Kras mutations in lung carcinomas. Mol Med. Rep 5, 599–602 (2012). 22200795 10.3892/mmr.2011.736

[b5] Gambhir S. S. Molecular imaging of cancer with positron emission tomography. Nat. Rev. Cancer 2, 683–693 (2002). 12209157 10.1038/nrc882

[b6] Niu G. & Chen X. Why integrin as a primary target for imaging and therapy. Theranostics 1, 30–47 (2011). 21544229 10.7150/thno/v01p0030 PMC3086610

[b7] Cai W. & Chen X. Multimodality molecular imaging of tumor angiogenesis. J. Nucl. Med.. 49 Suppl 2, 113S–28S (2008). 18523069 10.2967/jnumed.107.045922

[b8] Backer M. V. & Backer J. M. Imaging key biomarkers of tumor angiogenesis. Theranostics 2, 502–515 (2012). 22737188 10.7150/thno.3623 PMC3364556

[b9] Beer A. J., Kessler H., Wester H. J. & Schwaiger M. PET Imaging of Integrin alphaVbeta3 Expression. Theranostics 1, 48–57 (2011). 21547152 10.7150/thno/v01p0048 PMC3086612

[b10] Lee J. . RGD peptide-conjugated multimodal NaGdF4:Yb3+/Er3+ nanophosphors for upconversion luminescence, MR, and PET imaging of tumor angiogenesis. J. Nucl. Med. 54, 96–103 (2013). 23232276 10.2967/jnumed.112.108043

[b11] Haubner R. . Noninvasive visualization of the activated alphavbeta3 integrin in cancer patients by positron emission tomography and [18F]Galacto-RGD. PLoS Med. 2, e70 (2005). 15783258 10.1371/journal.pmed.0020070 PMC1069665

[b12] McParland B. J. . The biodistribution and radiation dosimetry of the Arg-Gly-Asp peptide 18F-AH111585 in healthy volunteers. J. Nucl. Med. 49, 1664–1667 (2008). 18794263 10.2967/jnumed.108.052126

[b13] Doss M. . Biodistribution and radiation dosimetry of the integrin marker 18F-RGD-K5 determined from whole-body PET/CT in monkeys and humans. J. Nucl. Med. 53, 787–795 (2012). 22499613 10.2967/jnumed.111.088955 PMC7607669

[b14] Mittra E. S. . Pilot pharmacokinetic and dosimetric studies of (18)F-FPPRGD2: a PET radiopharmaceutical agent for imaging alpha(v)beta(3) integrin levels. Radiology 260, 182–191 (2011). 21502381 10.1148/radiol.11101139 PMC3121013

[b15] Liu S. . 18F-labeled galacto and PEGylated RGD dimers for PET imaging of alphavbeta3 integrin expression. Mol Imaging Biol 12, 530–538 (2010). 19949981 10.1007/s11307-009-0284-2 PMC2999579

[b16] Guo J. . Comparison of three dimeric 18F-AlF-NOTA-RGD tracers. Mol Imaging Biol 16, 274–283 (2014). 23982795 10.1007/s11307-013-0668-1

[b17] Gao H. . PET imaging of angiogenesis after myocardial infarction/reperfusion using a one-step labeled integrin-targeted tracer 18F-AlF-NOTA-PRGD2. Eur. J. Nucl. Med. Mol. Imaging 39, 683–692 (2012). 22274731 10.1007/s00259-011-2052-1 PMC3319105

[b18] Chen X. . Integrin alpha v beta 3-targeted imaging of lung cancer. Neoplasia 7, 271–279 (2005). 15799827 10.1593/neo.04538 PMC1501139

[b19] Fushiki H. . Pre-clinical validation of orthotopically-implanted pulmonary tumor by imaging with 18F-fluorothymidine-positron emission tomography/computed tomography. Anticancer Res. 33, 4741–4749 (2013). 24222108

[b20] Metz S. . Phenotyping of tumor biology in patients by multimodality multiparametric imaging: relationship of microcirculation, alphavbeta3 expression, and glucose metabolism. J. Nucl. Med. 51, 1691–1698 (2010). 20956471 10.2967/jnumed.110.077719

[b21] Sauter A. W. . Multifunctional profiling of non-small cell lung cancer using 18F-FDG PET/CT and volume perfusion CT. J. Nucl. Med. 53, 521–529 (2012). 22414637 10.2967/jnumed.111.097865

[b22] Campanile C. . Characterization of different osteosarcoma phenotypes by PET imaging in preclinical animal models. J. Nucl. Med. 54, 1362–1368 (2013). 23801674 10.2967/jnumed.112.115527

[b23] Ekmekcioglu O. . Correlation of 18F-fluorodeoxyglucose uptake with histopathological prognostic factors in breast carcinoma. Nucl Med. Commun 34, 1055–1067 (2013). 24025919 10.1097/MNM.0b013e3283658369

[b24] Yen T. C. . 18F-FDG uptake in squamous cell carcinoma of the cervix is correlated with glucose transporter 1 expression. J. Nucl. Med. 45, 22–29 (2004). 14734665

[b25] Kurokawa T. . Expression of GLUT-1 glucose transfer, cellular proliferation activity and grade of tumor correlate with [F-18]-fluorodeoxyglucose uptake by positron emission tomography in epithelial tumors of the ovary. Int. J. Cancer 109, 926–932 (2004). 15027127 10.1002/ijc.20057

[b26] Lee D. W. . Role of SUVmax and GLUT-1 Expression in Determining Tumor Aggressiveness in Patients With Clinical Stage I Endometrioid Endometrial Cancer. Int. J. Gynecol. Cancer 25, 843–849 (2015). 25347093 10.1097/IGC.0000000000000301

[b27] Yan S. X. . Effect of antisense oligodeoxynucleotides glucose transporter-1 on enhancement of radiosensitivity of laryngeal carcinoma. Int J Med. Sci 10, 1375–1386 (2013). 23983599 10.7150/ijms.6855 PMC3753417

[b28] Michielsen K. . Whole-body MRI with diffusion-weighted sequence for staging of patients with suspected ovarian cancer: a clinical feasibility study in comparison to CT and FDG-PET/CT. Eur Radiol 24, 889–901 (2014). 24322510 10.1007/s00330-013-3083-8

[b29] Mi B. . Pilot Prospective Evaluation of (18)F-Alfatide II for Detection of Skeletal Metastases. Theranostics 5, 1115–1121 (2015). 26199649 10.7150/thno.12938 PMC4508500

[b30] Diederichs C. G., Staib L., Glatting G., Beger H. G. & Reske S. N. FDG PET: elevated plasma glucose reduces both uptake and detection rate of pancreatic malignancies. J. Nucl. Med. 39, 1030–1033 (1998). 9627339

[b31] Day C. P., Carter J., Bonomi C., Hollingshead M. & Merlino G. Preclinical therapeutic response of residual metastatic disease is distinct from its primary tumor of origin. Int. J. Cancer 130, 190–199 (2012). 21312195 10.1002/ijc.25978 PMC3161145

[b32] Gao D. . Endothelial progenitor cells control the angiogenic switch in mouse lung metastasis. Science 319, 195–198 (2008). 18187653 10.1126/science.1150224

[b33] Wan W. . First experience of 18F-alfatide in lung cancer patients using a new lyophilized kit for rapid radiofluorination. J. Nucl. Med. 54, 691–698 (2013). 23554506 10.2967/jnumed.112.113563 PMC3683452

